# Clinical Profile and Outcome of Japanese Encephalitis in Children Admitted with Acute Encephalitis Syndrome

**DOI:** 10.1155/2013/152656

**Published:** 2013-12-29

**Authors:** Gitali Kakoti, Prafulla Dutta, Bishnu Ram Das, Jani Borah, Jagadish Mahanta

**Affiliations:** ^1^Regional Medical Research Centre, ICMR, Northeast Region, P.O. Box No. 105, Dibrugarh, Assam 786 001, India; ^2^Department of Community Medicine, Assam Medical College, Dibrugarh Assam 786002, India

## Abstract

Japanese encephalitis (JE) is an arthropod borne viral disease. Children are most commonly affected in Southeast Asian region showing symptoms of central nervous system with several complications and death. The clinical characteristics and outcomes in pediatric JE patients hospitalized with acute encephalitis syndrome (AES) are still poorly understood. A prospective study was conducted in pediatric ward of Assam Medical College Hospital to evaluate the clinical profile and outcome of JE in children. A total of 223 hospitalized AES cases were enrolled during March to December 2012. Serum and cerebro spinal fluids were tested for presence of JE specific IgM antibody. 67 (30%) were found to be JE positive. The most common presenting symptoms in JE patients were fever (100%), altered sensorium (83.58%), seizure (82.08%), headache (41.79%), and vomiting (29.85%). Signs of meningeal irritation were present in 55.22% of cases. Around 40.29%, JE patients had GCS ≤ 8. Among the JE patients, 14.7% died before discharge. The complete recoveries were observed in 63.9% of cases, while 21.3% had some sort of disability at the time of discharge. JE is still a major cause of AES in children in this part of India. These significant findings thus seek attentions of the global community to combat JE in children.

## 1. Introduction

Japanese encephalitis (JE) is the most prevalent and significant mosquito borne viral encephalitis of man, occurring with an estimated 30,000 to 50,000 of cases and 15,000 deaths annually [1–3]. About 20% to 30% of JE cases are fatal, and 30–50% result in permanent neuropsychiatric sequelae [3, 4]. Children remain the main victims of the disease [5, 6]. In India, nearly all states have reported JE cases except that of Jammu & Kashmir, Himachal Pradesh, and Uttaranchal [7]. The Northeastern region (NE region) of India, particularly the upper part of the state of Assam, has been experiencing recurrent episodes of JE with different magnitudes from July to October every year [8]. Most JE infections are asymptomatic, and the ratio of symptomatic to asymptomatic infections ranges from 1 in 300 to 1 in 1000 [9, 10]. Japanese encephalitis virus (JEV) targets the central nervous system, clinically manifesting with fever, headache, vomiting, signs of meningeal irritation, and altered consciousness [11]. At present, there is no specific agent available against JE. Treatment of JE is therefore essentially symptomatic and intensive supportive care is important to avoid neurological sequelae [12].

This study was undertaken for a better understanding and to determine the clinical profile and outcome of JE in children hospitalized with AES cases which may help in early diagnosis and initiating prompt supportive care.

## 2. Materials and Methods

### 2.1. Case Enrollment and Sample Collection

All the hospitalized AES cases up to 12 years of age in pediatric ward of Assam Medical College hospital were included in this study. This is a tertiary level hospital and provides health care services to mainly seven districts of upper Assam and neighboring state Arunachal Pradesh and Nagaland. Most patients are referred to this apex level institute from periphery for better supportive care and treatment. The study was carried out during March to December 2012.

For investigating AES cases, WHO case definition was adopted. Clinically a case of AES is defined as fever or recent history of fever with change in mental status (including confusion, disorientation, coma, or inability to talk) and/or new onset of seizures (excluding simple febrile seizures). Other early clinical findings could include an increase in irritability, somnolence or abnormal behavior greater than that seen with usual febrile illness [13, 14]. All enrolled cases were worked up with the help of a predesigned and pretested proforma. After getting written informed consent 2 mL of blood and CSF samples were collected in sterile condition. The samples were then transferred under cold chain to Regional Medical Research Centre Laboratory, ICMR, Dibrugarh and stored at −80°C for further analysis. Reports of CSF samples analyzed for physical, chemical, and cytological examination and other relevant investigations done at the time of admission were recorded from the bed head tickets of the patient.

The study was approved by the Institutional Ethics Committee (Human) of Regional Medical Research Centre (ICMR), Dibrugarh, Assam, India.

### 2.2. Outcome of JE Cases

The outcome of the patients were recorded at the time of discharge. Few patients were released from the hospital against medical advice and their condition could not be assessed. They were disqualified from the outcome analysis. Outcome was defined as recovered completely, recovered with neurological sequelae, and death.

Neurological sequelae were defined by the presence of one or more of the following at discharge; impaired consciousness, weakness (monoparesis, hemiparesis, and quadriparesis), focal or generalized abnormal limb tone (hypertonia and hypotonia), focal or generalized abnormal limb reflexes (hyperreflexia and hyporeflexia), diagnosis of new onset or recurrent seizures, or new or recurrent extra pyramidal movement disorders [15].

### 2.3. Serology

JE virus specific IgM antibodies were detected by IgM antibody capture-enzyme-linked immunosorbent assay kits obtained from the National Institute of Virology (NIV), Pune, India. The test was standardized and reported by NIV in 1984 [16]. The performance of the test was evaluated by Christian Medical College (CMC), Vellore in 2002 [17]. The JE IgM kit contains all ready to use reagents and has also been evaluated by Centers for Disease Control (CDC), Fort Collins, CO, USA for its performance. Using the United States' CDC results as the reference standard, the NIV kit had sensitivity in CSF 75%, Serum 71%, and specificity 96% in CSF and 77% in Serum [18]. Serological cross-reactions are common within the flaviviruses that is, Dengue, Japanese encephalitis, and West Nile encephalitis [16, 19] and all are prevalent in this part of the country [20–22]. Therefore, the JEV-IgM positive samples were further tested for the presence of IgM antibody against other flaviviruses namely Dengue and West Nile by using Dengue IgM capture ELISA kit obtained from NIV, Pune, India and PanBio WNV IgM capture ELISA kit (Australia).

### 2.4. Statistical Analysis

Results were presented in the form of percentages, mean ± SD. Statistical association were analyzed with the help of chi square test and Fisher's exact test whichever was applicable.

## 3. Results

### 3.1. Demographic Characteristics

A total number of 223 hospitalized pediatric patients with AES were enrolled in the present study. Of which 67 patients (30%) were JE and 156 patients (70%) were non-JE. The JE cases were confirmed following detection of JEV specific IgM antibody either in CSF or serum. All the samples were found to be negative for the presence of IgM antibody against other flaviviruses, namely, Dengue and West Nile prevalent in this region. Among the JE positive patients 18 were diagnosed by only serum testing positive for anti-JEV IgM antibodies and 4 were identified following detection of anti-JEV IgM antibodies in CSF only. In 45 AES patients both serum and CSF were positive for JEV specific IgM antibody. Among the JE positive cases 32 (47.6%) were male and 35 (52.2%) were female. The predominant age group affected was 5 to 12 years (Table 1) and the youngest child affected was 5 months old. Majority of the patients (90%) were from the rural area and belonged to low socioeconomic group (63%). Most of the children (80.5%) were not vaccinated against JE. Vaccination status of 7.5% children was not known. However, only (11.9%) of the care giver could confirm that their children were vaccinated against JE by SA-14-14-2 during 2006–2010 mass vaccination campaign in Assam.

### 3.2. Clinical Profile

The clinical profile of JE positive patients was presented in Table 2. Patients with JE were presenting vivid signs of AES. The most common presenting symptoms recorded were moderate to high grade fever (100%), altered sensorium (83.58%), seizures (82.08%), headache (41.79%), and vomiting (29.85%). Signs of meningeal irritation were present in 55.22% of cases. Around 40.29% of JE patients had Glasgow comma scale (GCS) within 3 to 8. All the JE patients were presented to the hospital between 1 to 12 days from the onset of illness. Only one patient was admitted on 31st day from the onset.

### 3.3. Laboratory Parameters

The CSF WBC counts of the 67 patients ranged from 2.0/mm^3^ to 520.0/mm^3^ (42.63 ± 82.11). Elevated levels of WBC (>5/mm^3^) were found in 47 (77%) patients and predominantly lymphocytic in nature. The mean CSF protein and glucose level were 57.0 ± 27.2 mg/dL and 45.6 ± 12.4 mg/dL, respectively. Of these 32 (52.5%) had elevated (>40 mg/dL) level of protein.

### 3.4. Outcome of JE Patients

Outcome at discharge was recorded for 61/67 patients. The outcome of 6 (8.9%) patients could not be observed as they left the hospital against medical advice. Among the available 61 confirmed JE patients, 39 (63.9%) were recovered completely, while 13 (21.3%) cases had neurological sequelae at the time of discharge. 9 (14.7%) patient died in the hospital (Table 3).

The average duration of illness prior to the admission was 5.4 days. Fatality rate was more (17.95%) in children admitted less than 7 days from the onset of illness. However, it was not found to be statistically significant. The mortality was significantly more in patients with GCS between 3 to 8 (26.92% *P* < 0.05). Presences of meningeal signs were not found to be associated with fatal outcome. Similarly, no significant association was observed between high cell counts, elevated level of protein in CSF, and children fatality (Table 4).

## 4. Discussion

The present study demonstrates that JE is one of the leading forms of viral encephalitis of children in this part of the country. Around 30% of hospitalized children with AES were diagnosed as confirmed JE. Similar study carried out in Cuddalore district, Tamil Nadu also reported 29.3% patients with JE in hospitalized AES children [23]. In our study, children mostly affected were from rural areas (90%) and belong to low socioeconomic group (63%). This correlated well with the earlier studies where the patients were children of farmers or farm laborers of low socioeconomic group residing in rural areas [1, 24]. This may be due to favorable epidemiological factors like presence of water logged paddy field supporting profuse breeding of vector mosquitoes, piggeries in close proximity to residence, nonuse of bed nets and outdoor playing habits of children.

The age group mainly affected was 5 to 12 years and the youngest one was 5 months old. Majority of the affected children were not vaccinated (80.5%). Study of vaccination status of the affected children revealed some striking findings. Currently in Assam, JE vaccination with live vaccine (SA-14-14-2) has been included in routine immunization programme as per National Immunization Schedule (NIS). Previously to clear the backlog in children 1–15 years of age mass vaccination programme was conducted in 11 JE endemic districts of Assam in a phasewise manner since May 2006. However, it was evident from the present study that the vaccination programme could not cover the target children adequately. It has also been noticed from the cases documented in Assam Medical College Hospital registers that the prevalence of JE infection was high amongst the hospitalized AES children in 2006–2010. The highest prevalence was 49.6% in 2006, while lowest was 32.5% in 2008. In other years prevalence was fluctuated as 48.8% in 2007, 37% in 2009, and 41.7% in 2010. In contrary, JE incidences have been declining sharply in pediatric age group in Taiwan after the vaccination programme began in 1967 [25]. This emphasizes the need of quality coverage of JE mass vaccination program and consequently vaccination campaign should be evaluated for appropriate corrective measures [18]. Moreover, continuation of JE vaccination of children in routine immunization in these JE endemic districts of Assam should be a public health priority.

Among the clinical presentation, fever, altered sensorium, seizures, headache, and vomiting was the most common symptoms observed in this study. In children similar manifestation was also noted in earlier studies [25, 26]. Signs of meningeal irritation were frequently observed in more than half of the study patients as recorded in other studies [1, 27].

Elevated cell count (>5 cell/mm^3^) in CSF was noted in 77% of patients with lymphocytic predominance and elevated CSF protein level (>40 mg/dL) was recorded in 52.5% of study children. However, in a study by Avabratha et al. observed elevated cell count in 45.06% and protein in 74.67% study patients [26].

In our study, 21.13% JE patients had neurological sequelae at the time of discharge, while 14.7% had died in hospital. Mortality was associated with GCS within 3 to 8. Neurological sequelae in JE are the common observation [26, 28]. In a study of certain prognostic features in 49 patients of JE in Thailand only deep coma was found to correlate with mortality which is in conformity to the present study. Similar association was also noted in other different studies [26, 29–31]. We could not establish any association of mortality with the meningeal signs and elevated level of CSF cell count and CSF protein. In contrary to this, the study conducted by Avabratha et al. in Bellary, Karnataka, revealed association between mortality and meningeal signs [26]. This may be mentioned here that our observation is only from a small number of patients who died in hospital and it is an ongoing study. In future with more number of patients we may be able to shed some more light on mortality and its association with meningeal signs.

In conclusion, JE is still a major cause of AES in children in this part of India. The most common clinical presentations were fever, altered sensorium, seizure, headache, vomiting and signs of meningeal irritation. The case fatality rate was recorded as high as 14.7% due to JE in children admitted with AES. These significant research findings seek the attentions of the global community to combat the menace of this arboviral encephalitis in saving the life of children.

## Figures and Tables

**Table 1:** The demographic profile of JE patients.

Parameter	Number of patients (*n* = 67)	Percentage (%)
Age in years		
<1	2	2.98
1 to 5	21	31.34
5 to 12	44	65.67
Sex		
Male	32	47.76
Female	35	52.24
Settings		
Urban	7	10.44
Rural	60	89.56

**Table 2:** Clinical profile of children with JE (*n* = 67).

Features	Number	Percentage (%)
Fever	67	100
Altered sensorium	56	83.58
Headache	28	41.79
Irritable	3	4.47
Vomiting	20	29.85
Abnormal behavior	2	2.98
Diarrhoea	3	4.47
Seizure	55	82.08
Glasgow coma scale (GCS) ≤ 8	27	40.29
Signs of meningeal irritation	37	55.22

**Table 3:** Outcome of children with JE at the time of discharge.

Outcome	JE patients* (*n* = 61)	Percentage (%)
Recovered completely	39	63.9
Recovered with neurological sequelae	13	21.3
Death	9	14.7

*JE patient who left the health facility against medical advice was not included in outcome analysis.

**Table 4:** Association of independent variables and outcome of children with JE.

Symptoms/variables	Total patients (*n* = 61)	Fatal	Percentage (%)	Significance
(1) Age(years)				
<1	2	0	0	NS
1 to 5	17	2	11.76	
5 to 12	42	7	16.66	
(2) Gender				
Male	29	4	13.79	NS
Female	32	5	15.63	
(3) Duration of illness prior to admission				
<7 days	39	7	17.95	NS
≥7 days	13	2	15.38	
(4) GCS				
3 to 8	26	7	26.92	S
(5) Meningeal signs	33	6	18.18	NS
(6) CSF cell count, cell/mm^3^ >5 cells/mm^3^	47	5	10.64	NS
(7) CSF protein concentration >40 mg/dL	32	4	12.5	NS

NS: Not significant, S: Significant at 95% level.
